# Prognostic Value of Cerebrospinal Fluid and Serum Neurofilament Light Chain in Amyotrophic Lateral Sclerosis: A Correlation Study

**DOI:** 10.1002/brb3.70256

**Published:** 2025-01-26

**Authors:** Siqi Dong, Xiaoni Liu, Yanni Zhou, Jiatong Li, Zihan Qi, Zihan Wang, Wenbo Yang, Xiangjun Chen

**Affiliations:** ^1^ Department of Neurology, Huashan Hospital Fudan University Shanghai People's Republic of China; ^2^ National Center for Neurological Disorders Shanghai People's Republic of China; ^3^ Department of Anthropology and Human Genetics, School of Life Sciences Fudan University Shanghai People's Republic of China; ^4^ Human Phenome Institute Fudan University Shanghai People's Republic of China

**Keywords:** amyotrophic lateral sclerosis, biomarker, diagnosis, neurofilament light chain, prognosis

## Abstract

**Background:**

The diagnostic and prognostic values of serum neurofilament light chain (sNfL), in comparison to cerebrospinal fluid (CSF) neurofilament light chain (cNfL), and other clinical parameters in amyotrophic lateral sclerosis (ALS) at the time of diagnosis remain elusive.

**Methods:**

We examine paired serum and CSF samples from 80 ALS patients and 21 control subjects, all obtained at the time of diagnosis. Additional serum samples were collected from 51 other ALS patients. NfL concentrations were quantified using the single molecule array (Simoa) technique.

**Results:**

Our findings demonstrate a robust correlation between NfL levels in matched CSF and serum samples. Notably, both sNfL (*p* < 0.0001) and cNfL (*p* < 0.0001) exhibited significantly elevated levels in ALS patients compared to controls. Furthermore, baseline sNfL concentrations, as well as cNfL levels, emerged as predictive indicators of subsequent disease progression rate (sNfL: *p* < 0.0001, cNfL: *p* = 0.0005) and overall survival (sNfL: *p* = 0.0073, cNfL: *p* = 0.0044). Employing a Cox regression model, we identified baseline sNfL level (HR = 1.01, *p* = 0.013), and diagnostic delay (HR = 0.94, *p* = 0.003) as independent prognostic factors for mortality. Furthermore, we constructed a nomogram model that incorporates both sNfL and pertinent clinical variables, which substantially enhances the accuracy of predicting disease outcomes (Concordance Index, 0.808).

**Conclusion:**

Our study underscores the robust correlation between sNfL and cNfL in ALS patients and establishes baseline sNfL as a potent and independent prognostic marker for mortality.

## Graphical abstract

This study evaluates the diagnostic and prognostic value of serum (sNfL) and cerebrospinal fluid neurofilament light chain (cNfL) levels in amyotrophic lateral sclerosis (ALS) patients. Analyzing 80 paired samples revealed a strong correlation between sNfL and cNfL, with sNfL demonstrating an AUC of 0.9635, sensitivity of 96.12%, and specificity of 90.48% at a cutoff of >19.12 pg/mL. An individualized prognostic nomogram was developed using sNfL and clinical variables, supporting sNfL as a reliable, noninvasive biomarker for ALS diagnosis, reducing the need for invasive cerebrospinal fluid analysis.

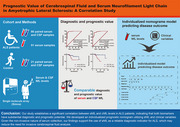

## 1 Introduction

Neurofilament light chain (NfL) has emerged as a promising diagnostic and prognostic biomarker for amyotrophic lateral sclerosis (ALS). Previous studies, including recent findings by Halbgebauer et al., have demonstrated the potential of both cerebrospinal fluid (CSF) NfL (cNfL) and serum NfL (sNfL) as effective diagnostic markers, capable of distinguishing ALS from its mimics with high sensitivity and specificity (Halbgebauer et al. 2022; Feneberg et al. 2017; Verde et al. 2019; Li et al. 2018). While the research conducted by Halbgebauer et al. offers important insights into the comparative analysis of sNfL and cNfL, their study was conducted only in Caucasian populations and did not analyze the association between NfL levels and other clinical parameters. Further studies are needed to expand their conclusions.

Importantly, elevated NfL levels have been consistently associated with more rapid disease progression and shorter survival in ALS patients (Lu et al. 2015; Gaiani et al. 2017; Benatar et al. 2020; Zhou et al. 2021; Thouvenot et al. 2020). Despite the compelling evidence regarding the utility of NfL, the comparative diagnostic and prognostic value of sNfL versus cNfL at the time of ALS diagnosis remains to be fully elucidated. While some studies suggest that cNfL exhibits superior diagnostic accuracy (Feneberg et al. 2017; Lu et al. 2015) and outperforms sNfL in predicting disease trajectory (Lu et al. 2015; Weydt et al. 2016), the relative noninvasive nature of serum acquisition raises important questions regarding whether sNfL could serve as a reliable alternative indicator of ALS diagnosis and prognosis.

In this study, we obtained paired serum and cerebrospinal fluid samples from 80 ALS patients and 21 control subjects at the time of diagnosis. Additional serum samples were collected from 51 other ALS patients. NfL concentrations were quantified using the single molecule array (Simoa) technique. Our investigation focuses on exploring the diagnostic and prognostic value of both sNfL and cNfL. Additionally, we aim to construct an integrated prognostic model that incorporates sNfL and relevant clinical variables.

## 2 Materials and Methods

### 2.1 Participants and Sampling

In this study, we recruited individuals diagnosed with ALS in accordance with the revised El Escorial criteria between July 2018 and December 2022 at Huashan Hospital, Fudan University. The study included a total of 131 unrelated patients of Chinese descent, as outlined in Table 1. As control subjects, we enrolled 21 individuals with neuropsychiatric symptoms, including hysteria (5 patients), schizophrenia (4 patients), headache (3 patients), cervical spondylosis (3 patients), facial paralysis (3 patients), peripheral neuropathy (2 patients), and ankylosing spondylitis (1 patient). For analysis, paired serum and CSF samples were obtained from 80 ALS patients and 21 control subjects at the time of diagnosis. Additionally, serum samples were collected from an additional 51 ALS patients. All participating subjects provided written informed consent, and the study protocol was approved by the Ethic Committee of Huashan Hospital, Fudan University. To ensure accurate diagnosis, differential diagnosis was conducted by specialized neurologists based on clinical presentation, laboratory testing, genetic testing, and magnetic resonance imaging (MRI), aiming to exclude conditions that may mimic ALS, including autoimmune diseases. Motor assessments, including the ALS Functional Rating Scale–Revised (ALSFRS‐R), were performed by skilled neurologists. The patients were then followed up via telephone or during their return visits until June 2023.

**TABLE 1:** Demographic characteristics of the cohort population.

Total ALS cases	CSF and serum	Serum only	Total
Self‐reported Han Chinese	80 (100%)	51 (100%)	131 (100%)
Male sex	38 (47.50%)	32 (62.75%)	70 (53.44%)
Bulbar onset	22 (27.50%)	17 (33.33%)	39 (29.77%)
Age at onset	56 (47–65)	53 (45–60)	54 (46–62)
Baseline BMI (kg/m^2^)	22.86 ± 2.39	22.91 ± 2.64	22.91 ± 2.48
Baseline FVC (%)	78.69 ± 25.45	84.67 ± 22.71	84.67 ± 22.71
Disease progression rate (/m)	0.98 ± 0.85	0.97 ± 0.64	0.93 ± 0.78
Survival (m)	20 (16.5–25.5)	28 (19.5–33.5)	25.5 (18.25–32.75)
Reaching the outcome	11 (13.75%)	27 (52.94%)	38 (29.01%)

### 2.2 NfL Quantification

After collecting the samples, they were centrifuged at 2000 g for 10 min at room temperature, and aliquots were stored at −80°C until further use. The concentration of NfL was determined using a commercially available NfL assay kit (Quanterix, USA) based on ultrasensitive single molecular assay (Simoa) technology. Each plate included calibrators spanning the range of 0–500 pg/mL for NfL, along with quality controls. Serum samples were four‐fold diluted, while CSF samples were 100‐fold diluted using the provided dilution buffer to ensure they fell within the range of the standard curve.

### 2.3 Statistical Analysis

Symptom onset was defined as the first occurrence of limb weakness, dysarthria, dysphagia, or dyspnea. The disease progression rate (DPR) was defined as ALSFRS‐R slopes during intervention (loss of points per month) which were calculated by linear regression based on 3‐monthly evaluations for each patient. Survival duration was defined as time from symptom onset to permanent assisted ventilation (PAV) (≥ 22 h/d noninvasive ventilation), tracheostomy, or death. BMI prior to disease onset was calculated according to World Health Organization (WHO) specifications. Patients with weight loss were defined those with BMI decline > 1 kg/m^2^ within a year of follow‐up.

Statistical analyses were performed using Graphpad Prism (v6.0) and R(v4.0.3). Data were reported as median (interquartile range) or mean ± standard deviation. Since most of the data did not follow a normal distribution, intergroup differences were assessed for significance using the unpaired Mann–Whitney U‐test. The diagnostic accuracy of NfL was assessed using receiver operating characteristic (ROC) curves, and an optimal cutoff was calculated using Youden's index. The association of sNfL, cNfL, and other clinical parameters was estimated by Spearman's rank correlation coefficient (*r*). The prognostic value of NfL was evaluated by Kaplan–Meier (univariate) and Cox regression (multivariate) methods. The nomogram was constructed using the training group dataset by rms package (version 6.3.0) in R. The prediction accuracy was evaluated by logistic calibration curves. C‐Index values were used to compare the predictive effect of survival models. All statistical tests were two‐tailed, with a significance threshold set to 0.05.

## 3 Results

### 3.1 Cross‐Sectional Analyses of NfL Levels in CSF and Serum

In this study, we conducted cross‐sectional analyses of NfL levels in CSF and serum. Table 1 presents the demographic characteristics of the 131 ALS subjects included in the analysis. Of these patients, paired serum and CSF samples were obtained at the time of diagnosis for 80 individuals, while serum samples alone were available for the remaining 51 patients. Our control group consisted of 21 individuals with nonneuroinflammatory conditions, including hysteria (5 patients), schizophrenia (4 patients), headache (3 patients), cervical spondylosis (3 patients), facial paralysis (3 patients), peripheral neuropathy (2 patients), and ankylosing spondylitis (1 patient).

**FIGURE 1:**
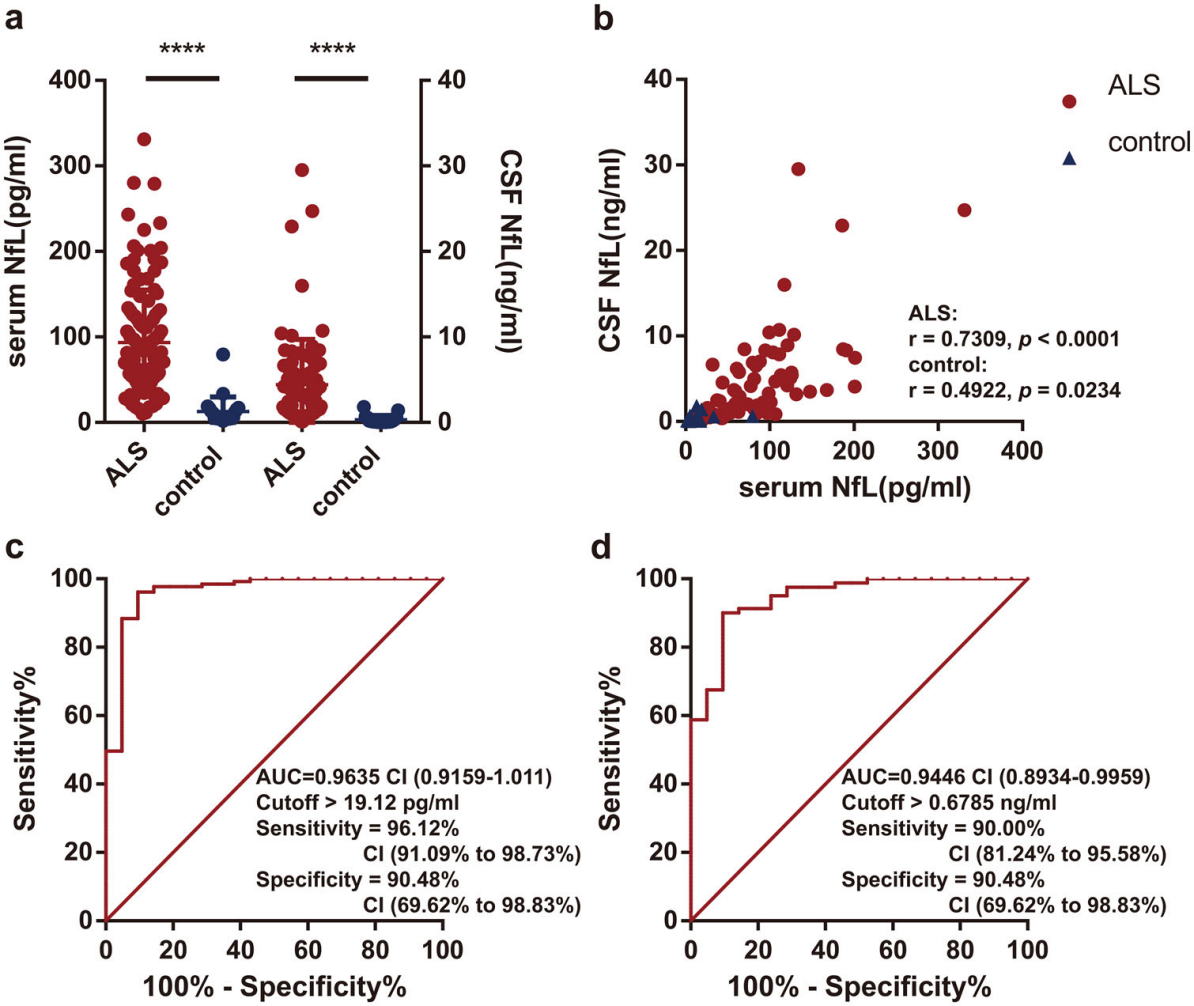
Comparison of serum and CSF NfL concentrations in ALS patients versus controls. NfL concentrations measured in serum (*n* = 131 for ALS; *n* = 21 for controls) and CSF (*n* = 80 for ALS; *n* = 21 for Controls) from patients with ALS compared to controls in a cross‐sectional analysis. (a) NfL concentrations in serum (left) and CSF (right). (b) Spearman's rank correlation between paired CSF and serum NfL levels in controls and ALS patients. (c) ROC analysis for serum NfL. (d) ROC analysis for CSF NfL.

Significantly elevated levels of both sNfL (*p* < 0.0001) and cNfL (*p* < 0.0001) were observed in ALS patients compared to controls. Furthermore, we found a strong correlation between NfL levels in matched CSF and serum samples, with a correlation coefficient (*r*) of 0.7309 (*p* < 0.0001) among ALS patients, and 0.4922 (*p* = 0.0234) among controls (Figure 1b). cNfL values were 36.2‐fold (interquartile range: 22.7–64.8) and 16.7‐fold (10.1–26.5) higher than serum levels in ALS and controls, respectively (*p* < 0.0001). Receiver operating characteristic (ROC) analysis demonstrated a comparable diagnostic value of sNfL and cNfL, yielding an area under the curve (AUC) of 0.9635 for sNfL and 0.9446 for cNfL (*p* < 0.0001) (Figure 1c,d). Notably, the defined cutoff levels were 19.12pg/mL for sNfL and 0.6785 ng/mL for cNfL, which effectively differentiated ALS patients from controls for both sNfL and cNfL (Figure 1c,d). For a further examination of the diagnostic potential of sNfL and cNfL, we restricted the dataset to patients who had contributed paired serum and cerebrospinal fluid samples (*n* = 80). Remarkably, sNfL maintained its diagnostic parity with cNfL, boasting an AUC of 0.9560.

### 3.2 Correlation Between NfL Concentrations and Clinical Variables

**FIGURE 2:**
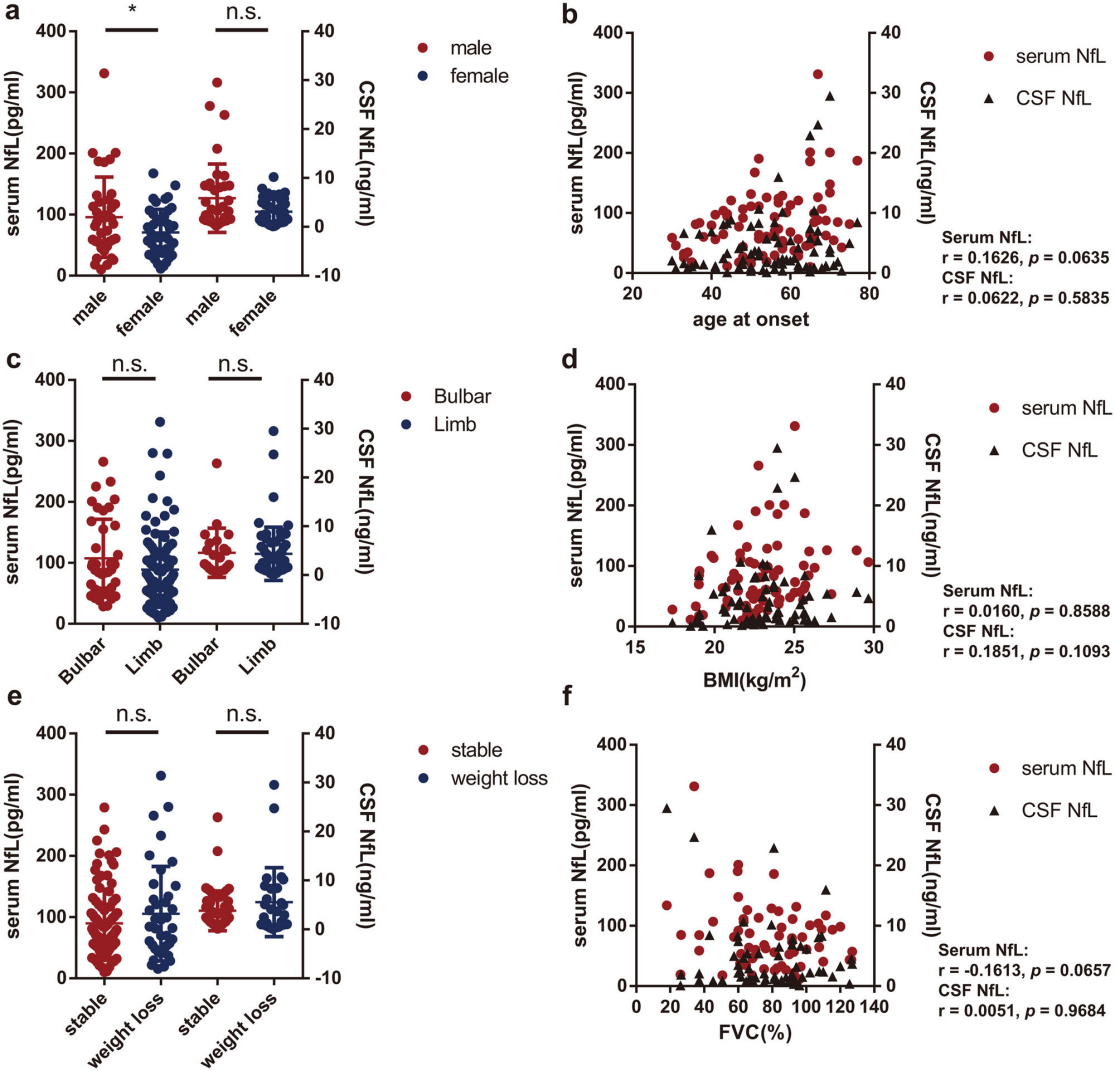
Correlation of NfL Concentrations with Clinical Variables in ALS. (a) Comparison of serum (left, *n* = 70 for male, *n* = 61 for female) and CSF (right, *n* = 38 for male, *n* = 42 for female) NfL levels between male and female ALS patients. (b) Spearman's correlation assessing the relationship between age at onset and NfL levels in serum (*n* = 131) and CSF (*n* = 80). (c) Serum (left, *n* = 39 for bulbar, *n* = 92 for limb) and CSF (right, *n* = 22 for bulbar, *n* = 58 for limb) NfL levels compared in patients with bulbar versus limb onset. (d) Correlation of baseline BMI with serum (sample size: *n* = 131) and CSF (sample size: *n* = 80). (e) Serum (left, *n* = 93 for stable, *n* = 38 for weight loss) and CSF (right, *n* = 52 for stable, *n* = 28 for weight loss) NfL levels assessed in patients with/without weight loss. (f) Spearman's correlation assessing the relationship between forced vital capacity (FVC) and NfL concentrations in serum (*n* = 131) and CSF (*n* = 80).

We conducted further evaluations to determine whether there existed any correlations between the concentration of NfL and various clinical variables, including sex, site of disease onset, age at onset, baseline BMI, the rate of BMI decline per month, and baseline forced vital capacity (FVC). We observed that sNfL levels were elevated in male patients (*p* = 0.0492, Figure 2a). However, we did not find any significant correlation between sNfL and the site of disease onset (*p* = 0.6742, Figure 2c), baseline BMI (*p* = 0.8588, Figure 2d) or weight loss(*p* = 0.5040, Figure 2e). Although not statistically significant, there was a trend suggesting that patients with an older age at onset (*r* = 0.1626, *p* = 0.0635, Figure 2b) and lower baseline FVC (*r* = −0.1636, *p* = 0.0657, Figure 2f) exhibited higher levels of sNfL.

When we narrowed down the dataset to the 80 individuals with paired serum and CSF samples, we found that sNfL remained significantly correlated with age at onset (*r* = 0.2693, *p* = 0.0157) and baseline BMI (*r* = 0.2809, *p* = 0.0133). Additionally, there was a tendency for sNfL to be higher in male patients (*p* = 0.1197) and negatively correlated with baseline FVC (*r* = −0.2001, *p* = 0.1159). However, cNfL did not exhibit significant correlations with any of the clinical variables (Figure 2), except for a tendency to be higher in male individuals (*p* = 0.1053, Figure 2a) or those with a higher baseline BMI (*r* = 0.1851, *p* = 0.1093, Figure 2d).

### 3.3 Prognostic Value of Baseline NfL Concentrations

**FIGURE 3:**
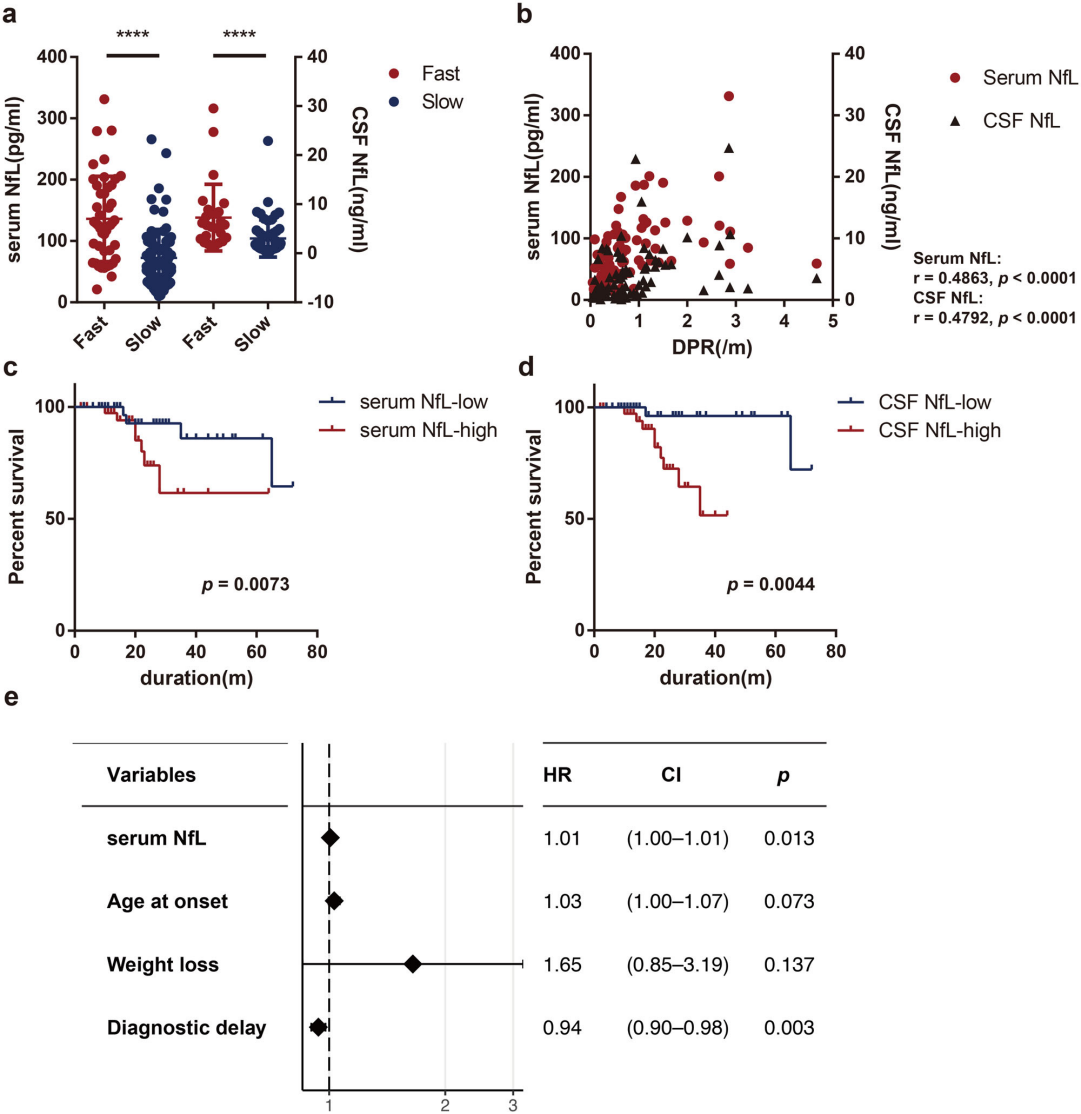
Association of serum and CSF NfL levels with disease progression in ALS. (a) Comparison of serum (left, *n* = 45 for fast, *n* = 86 for slow) and CSF(right, *n* = 26 for fast, *n* = 54 for slow) NfL levels in fast‐progressing versus slow‐progressing ALS patients. (b) Spearman's correlation between disease progression rate (DPR) and NfL levels in serum (*n* = 131) and CSF (*n* = 80). (c) Kaplan–Meier survival curves for serum NfL, using a median cutoff of 77.30 pg/mL, illustrating its prognostic significance. (d) Kaplan–Meier survival curves for CSF NfL with a median cutoff of 2.41 ng/mL. (e) Forest plot displaying the hazard ratio of the Cox proportional hazards model, with two‐sided 95% confidence intervals represented by the error bars.

We next compared the prognostic value of sNfL and cNfL. Patients were divided into fast‐progressing (DPR ≥ 1) and slow‐progressing (DPR < 1) groups based on disease progression rate (DPR). Both sNfL and cNfL levels were significantly higher in the fast‐progressing group (*p* < 0.0001, Figure 3a). Spearman's rank correlation test showed a significant correlation between NfL levels and DPR, comparable for sNfL and cNfL (sNfL: *r* = 0.4863, *p* < 0.0001; cNfL: *r* = 0.4792, *p* < 0.0001, Figure 3b). The Kaplan–Meier survival analysis demonstrated a significant association between higher sNfL and cNfL levels and a poorer prognosis (sNfL: *p* = 0.0073; cNfL: *p* = 0.0044, Figure 3c,d) when patients were stratified into low and high NfL groups based on the median sNfL or cNfL levels.

In the subset with paired serum and CSF samples (*n* = 80), sNfL remained significantly higher in the fast‐progressing group (*p* < 0.0001) and correlated strongly with DPR (*r* = 0.5610, *p* < 0.0001). Although not statistically significant, there was a trend suggesting poorer survival with higher sNfL levels (*p* = 0.0653).

In a cohort of patients with serum samples (*n* = 131), we conducted univariate Cox regression analysis to identify variables significantly associated with mortality. Our analysis indicated that baseline sNfL levels, age at onset, weight loss, and diagnostic delay were associated with increased mortality, while other variables—including sex, site of onset, BMI, FVC, alcohol consumption, smoking status, Riluzole use, and diagnostic category—were not significantly associated (Table 2). Subsequently, we performed multivariate Cox regression analysis, which identified baseline sNfL level (HR = 1.01, *p* = 0.013), and diagnostic delay (HR = 0.94, *p* = 0.003) as independent prognostic factors for mortality (Figure 3e). Notably, Cox regression analysis could not be performed for cNfL due to insufficient sample size.

**TABLE 2:** Univariate Cox regression analysis of sNFL and clinical variables.

Variable	HR (95% CI)	*p* value
sNfL	1 (1–1)	0.0000046
Male sex	2 (0.98–3.9)	0.056
Bulbar onset	0.77 (0.36–1.6)	0.5
Age at onset	1 (1–1.1)	0.028
Baseline BMI	1 (0.89–1.2)	0.81
Weight loss	2 (1.1–3.8)	0.032
Baseline FVC	0.98 (0.97–1)	0.057
Drink	1.3 (0.7–2.5)	0.39
Smoke	1.2 (0.62–2.2)	0.62
Riluzole use	1.2 (0.29–5)	0.81
Diagnostic delay	0.92 (0.88–0.96)	0.00023
Diagnostic category	1.5 (0.69–3.3)	0.3

### 3.4 Prognostic Nomogram Constructed With Serum NfL and Clinical Variables

**FIGURE 4:**
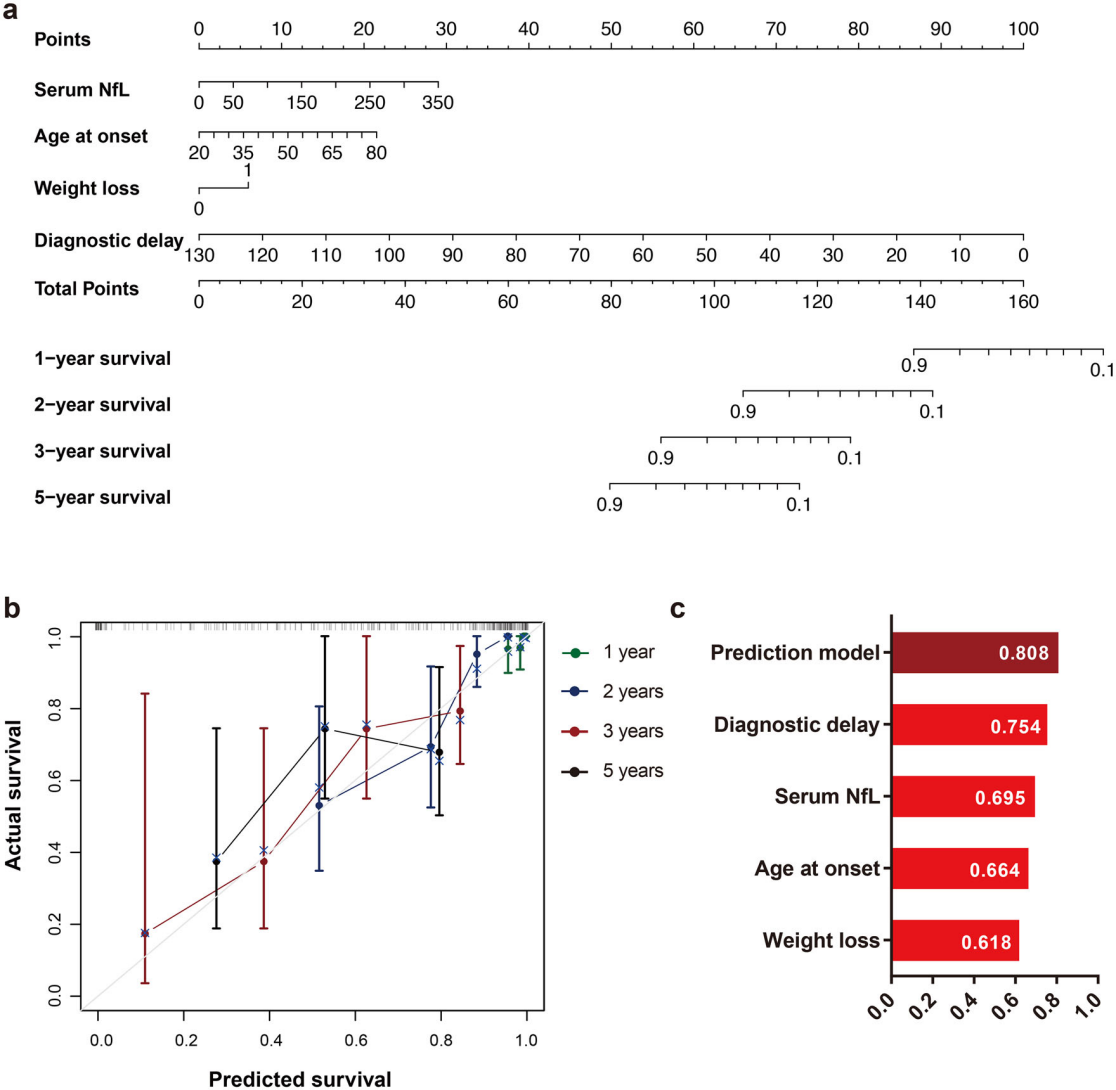
Individualized nomogram prediction model for ALS Survival. (a) Predicted 1‐, 2‐, 3‐, and 5‐year survival rates for ALS patients based on sNfL and clinical variables (sample size: *n* = 131). (b) Calibration plots comparing predicted versus actual survival probabilities over the same time intervals. (c) Evaluation of the model's predictive power via C‐Index, demonstrating this nomogram's capacity for individual survival prediction.

Next, we developed an individualized prognostic nomogram prediction model using disease‐related factors we identified by univariate Cox regression analysis, including baseline sNfL levels, age at onset, weight loss, and diagnostic delay. These significant factors were integrated into the nomogram prediction model, as depicted in Figure 4a. By assigning points to each independent factor and calculating their cumulative sum, a predictive probability was visually represented. To assess the performance of the developed nomogram, we examined its calibration curve. The findings revealed a substantial overlap between the nomogram predictions and the actual observed outcomes, indicating an acceptable level of predictive accuracy (Figure 4b). Furthermore, we calculated the C‐index of the nomogram model (Figure 4c). Among the individual clinical factors evaluated, only diagnostic demonstrated a C‐index value surpassing 0.7. Overall, the nomogram model, incorporating both sNfL and clinical variables, exhibited a C‐index of 0.808. This finding underscores the strong prognostic value provided by the combination of sNfL and clinical variables in predicting ALS outcomes (Figure 4c).

## 4 Discussion

Recent advancements in our understanding of neurofilaments, particularly the NfL, have identified them as significant diagnostic and prognostic biomarkers for ALS. Our study contributes to this growing body of literature by conducting a comprehensive comparison between sNfL and cNfL in ALS patients.

We demonstrate a robust correlation between sNfL and cNfL among ALS patients, reflected in a Spearman's correlation coefficient of *r* = 0.7309 (*p* < 0.0001). This finding aligns with prior studies (Halbgebauer et al. 2022; Lu et al. 2015; Thouvenot et al. 2020), reinforcing the potential of sNfL as an effective biomarker for ALS diagnosis and disease progression. The ROC analysis further supports this, revealing comparable diagnostic accuracies for both biomarkers, with AUC values of 0.9560 for sNfL and 0.9446 for cNfL.

Subsequently, we employed DPR and survival as outcome indicators to assess the prognostic values of sNfL and cNfL, respectively. It is noteworthy that DPR was defined as the slope of the ALSFRS‐R during intervention (i.e., loss of points per month). These values were calculated using linear regression based on 3‐monthly evaluations for each patient to enhance reliability (Benatar et al. 2020; Dorst et al. 2020). The results demonstrated promising diagnostic value for both sNfL and cNfL, whether assessed through DPR(Figure 3a,b) or Kaplan–Meier survival analysis (Figure 3c,d)

While previous work, especially the research conducted by Halbgebauer et al. offers important insights into the comparative analysis of sNfL and cNfL (Halbgebauer et al. 2022; Feneberg et al. 2017; Lu et al. 2015; Thouvenot et al. 2020), our study expands upon their findings by providing a more detailed analysis of the correlations between NfL levels and various clinical parameters, including sex, weight loss, and ALSFRS‐R progression. Moreover, we employed multivariate Cox regressions and an individualized prognostic nomogram incorporating sNfL and clinical variables. These analyses provide deeper insights into the prognostic value of sNfL.

One limitation of our study is the choice of controls. Our study included patients with individuals with neuropsychiatric symptoms rather than ALS‐mimicking conditions. Future research should consider a broader control group that encompasses ALS‐mimicking disorders to enhance the specificity of neurofilament levels in distinguishing ALS from other conditions. Additionally, nonmotor symptoms associated with ALS, such as cognitive (Elamin et al. 2011), behavioral (Nguyen et al. 2021), and autonomic dysfunctions (Dubbioso et al. 2023), warrant greater attention. Recent studies have indicated that these symptoms correlate with poorer outcomes in ALS patients (Elamin et al. 2011; Nguyen et al. 2021; Dubbioso et al. 2023). Addressing this dimension could lead to a more comprehensive understanding of ALS progression and the role of neurofilaments within that context.

## 5 Conclusion

In conclusion, our study establishes a significant correlation between sNfL and cNfL levels in ALS patients, indicating that both biomarkers have substantial diagnostic and prognostic potential. We developed an individualized prognostic nomogram utilizing sNfL and clinical variables. Given the noninvasive nature of serum collection, our findings support the use of sNfL as a reliable diagnostic indicator for ALS, which may reduce the need for invasive cerebrospinal fluid analysis.

## Author Contributions

**Siqi Dong**: methodology, software, data curation, formal analysis, validation, investigation, writing–original draft, visualization, conceptualization. **Xiaoni Liu**: methodology, data curation, formal analysis, validation, investigation, visualization, conceptualization, resources. **Yanni Zhou**: software, data curation, methodology, investigation, validation, formal analysis, visualization. **Jiatong Li**: investigation, data curation. **Zihan Qi**: investigation. **Zihan Wang**: data curation. **Wenbo Yang**: writing–review and editing, supervision, project administration, resources. **Xiangjun Chen**: writing–review and editing, conceptualization, supervision, project administration, funding acquisition, resources.

## Conflicts of Interest

The authors declare no conflicts of interest.

### Peer Review

The peer review history for this article is available at https://publons.com/publon/10.1002/brb3.70256.

## Data Availability

The data that support the findings of this study are available from the corresponding author upon reasonable request.

## References

[brb370256-bib-0001] Benatar, M. , L. Zhang , L. Wang , et al. 2020. “Validation of Serum Neurofilaments as Prognostic and Potential Pharmacodynamic Biomarkers for ALS.” Neurology 95, no. 1: e59–e69. 10.1212/WNL.0000000000009559. 32385188 PMC7371380

[brb370256-bib-0002] Dorst, J. , J. Schuster , J. Dreyhaupt , et al. 2020. “Effect of High‐Caloric Nutrition on Serum Neurofilament Light Chain Levels in Amyotrophic Lateral Sclerosis.” Journal of Neurology, Neurosurgery, and Psychiatry 91, no. 9: 1007–1009. 10.1136/jnnp-2020-323372. 32788256

[brb370256-bib-0003] Dubbioso, R. , V. Provitera , D. Pacella , et al. 2023. “Autonomic Dysfunction Is Associated With Disease Progression and Survival in Amyotrophic Lateral Sclerosis: A Prospective Longitudinal Cohort Study.” Journal of Neurology 270, no. 10: 4968–4977. 10.1007/s00415-023-11832-w. 37358634 PMC10511550

[brb370256-bib-0004] Elamin, M. , J. Phukan , P. Bede , et al. 2011. “Executive Dysfunction Is a Negative Prognostic Indicator in Patients With ALS Without Dementia.” Neurology 76, no. 14: 1263–1269. 10.1212/WNL.0b013e318214359f. 21464431

[brb370256-bib-0005] Feneberg, E. , P. Oeckl , P. Steinacker , et al. 2017. “Multicenter Evaluation of Neurofilaments in Early Symptom Onset Amyotrophic Lateral sclerosis.” Neurology 90, no. 1: e22–e30. 10.1212/WNL.0000000000004761. 29212830

[brb370256-bib-0006] Gaiani, A. , I. Martinelli , L. Bello , et al. 2017. “Diagnostic and Prognostic Biomarkers in Amyotrophic Lateral Sclerosis.” JAMA neurology 74, no. 5: 525. 10.1001/jamaneurol.2016.5398. 28264096 PMC5822207

[brb370256-bib-0007] Halbgebauer, S. , P. Steinacker , F. Verde , et al. 2022. “Comparison of CSF and Serum Neurofilament Light and Heavy Chain as Differential Diagnostic Biomarkers for ALS.” Journal of Neurology, Neurosurgery, and Psychiatry 93: 68–74. 10.1136/jnnp-2021-327129. 34417339

[brb370256-bib-0008] Li, D. , H. Ren , A. Jeromin , et al. 2018. “Diagnostic Performance of Neurofilaments in Chinese Patients With Amyotrophic Lateral Sclerosis: A Prospective Study.” Frontiers in Neurology 9: 726. 10.3389/fneur.2018.00726. 30210445 PMC6121092

[brb370256-bib-0009] Lu, C. H. , C. Macdonald‐Wallis , E. Gray , et al. 2015. “Neurofilament Light Chain: A Prognostic Biomarker in Amyotrophic Lateral Sclerosis.” Neurology 84, no. 22: 2247–2257. 10.1212/WNL.0000000000001642. 25934855 PMC4456658

[brb370256-bib-0010] Nguyen, C. , J. Caga , C. J. Mahoney , et al. 2021. “Behavioural Changes Predict Poorer Survival in Amyotrophic Lateral Sclerosis.” Brain and Cognition 150: 105710. 10.1016/j.bandc.2021.105710. 33725515

[brb370256-bib-0011] Thouvenot, E. , C. Demattei , S. Lehmann , et al. 2020. “Serum Neurofilament Light Chain at Time of Diagnosis Is an Independent Prognostic Factor of Survival in amyotrophic Lateral sclerosis.” European Journal of Neurology 27, no. 2: 251–257. 10.1111/ene.14063. 31437330

[brb370256-bib-0012] Verde, F. , P. Steinacker , J. H. Weishaupt , et al. 2019. “Neurofilament Light Chain in Serum for the Diagnosis of Amyotrophic Lateral Sclerosis.” Journal of Neurology, Neurosurgery, and Psychiatry 90, no. 2: 157–164. 10.1136/jnnp-2018-318704. 30309882

[brb370256-bib-0013] Weydt, P. , P. Oeckl , A. Huss , et al. 2016. “Neurofilament Levels as Biomarkers in Asymptomatic and Symptomatic Familial Amyotrophic Lateral sclerosis.” Annals of Neurology 79, no. 1: 152–158. 10.1002/ana.24552. 26528863

[brb370256-bib-0014] Zhou, Y. , Y. Chen , S. Dong , et al. 2021. “Role of Blood Neurofilaments in the Prognosis of Amyotrophic Lateral Sclerosis: A Meta‐Analysis.” Frontiers in Neurology 12: 712245. 10.3389/fneur.2021.712245. 34690913 PMC8526968

